# Artificial intelligence needs better health systems to reduce inequalities

**DOI:** 10.1038/s41746-026-03041-2

**Published:** 2026-07-17

**Authors:** Robin van Kessel, Alexander W. Carter, Khurram Nasir, Elias Mossialos

**Affiliations:** 1 AI and Digital Lab, LSE Health, Department of Health Policy, London School of Economics and Political Science, London, UK; 2 Digital Health Cluster, Hasso-Plattner-Institute, University of Potsdam, Potsdam, Germany; 3 Center for Cardiovascular Computational & Precision Health, DeBakey Heart & Vascular Center, Houston Methodist, Houston, TX USA; 4 Houston Methodist-Rice Digital Health Institute, Houston, TX USA

**Keywords:** Health care, Mathematics and computing, Medical research, Scientific community, Social sciences

## Abstract

Artificial Intelligence (AI) technologies are increasingly prevalent in healthcare, yet without adaptive governance, even well-designed systems risk exacerbating health inequalities. This perspective examines five interconnected governance domains: legal frameworks, evidence generation, regulation and market access, workforce readiness, and public trust. We argue these form a cyclical governance chain in which weaknesses cascade across domains, and identify critical gaps and system-level reforms to ensure AI reduces rather than amplifies disparities.

## Introduction

Artificial Intelligence (AI) technologies use algorithms or models to perform tasks and exhibit behaviours such as learning, making decisions, and making predictions. They are increasingly prevalent in society, including in an array of potential applications in health systems^1^. Concurrently, claims that AI technologies have the “ability to make healthcare more personalized, predictive, participatory, and preventative” and “make the performance of healthcare services more visible and monitorable” are yet to be actualised^2^. This rhetoric results from studies that show that AI technologies can improve on the current standard of care^3^, innovate health services delivery^4^, and enhance drug discovery processes^5^. The embedding of AI technologies coincides with their emergence as a major digital determinant of health, thereby shaping broader social, political, and economic systems^6^. There is a pressing need to examine AI applications in the context of incumbent systems (e.g., surveillance capitalism, the privatization of public healthcare infrastructures, and corporate consolidation). Without scrutiny, even AI systems that are designed following every best practice guideline can inadvertently reinforce existing power asymmetries and exacerbate inequalities^7,8^.

**Fig. 1:**
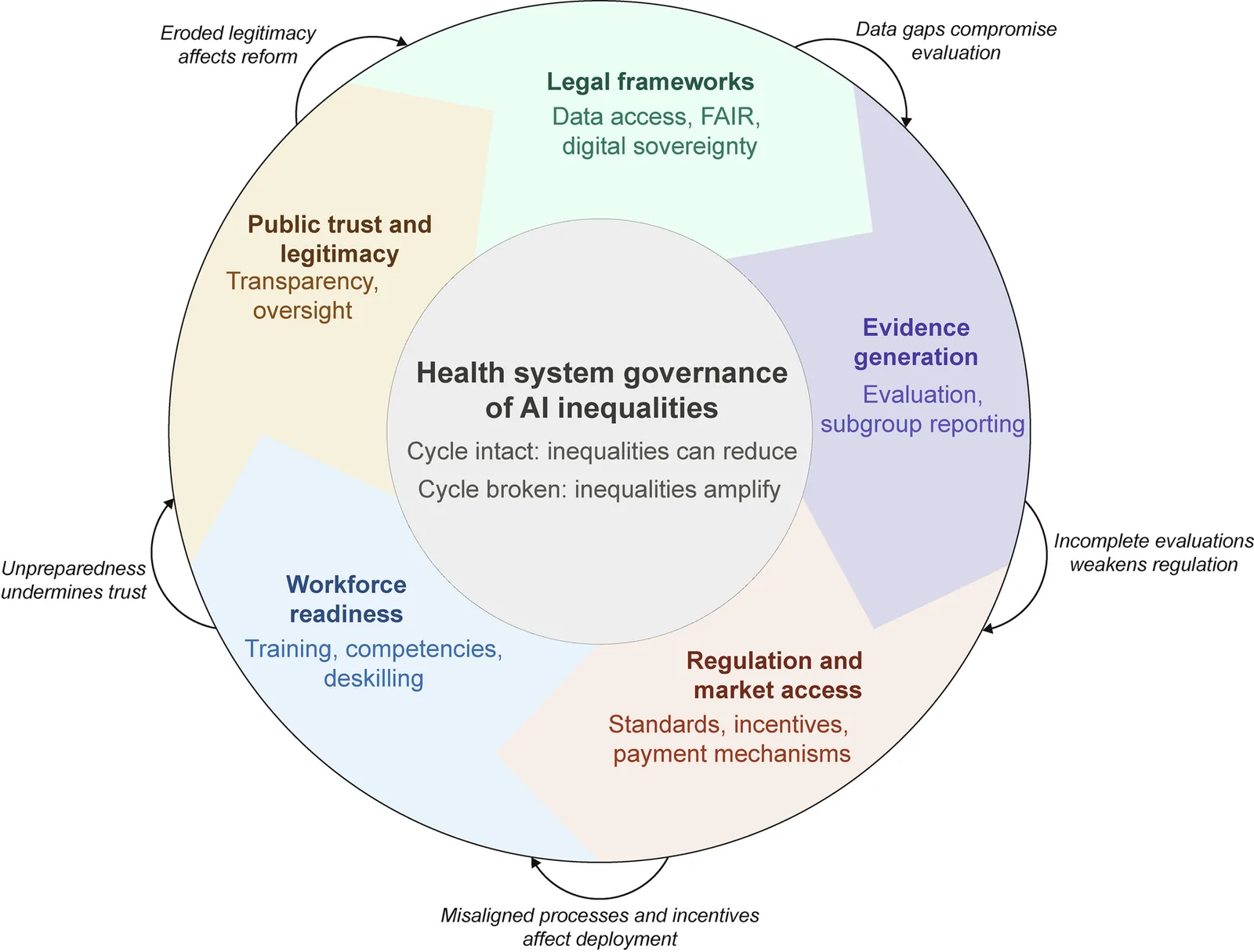
A cyclical depiction of the governance chain for AI inequalities in health systems. Five governance domains form a cyclical chain: legal frameworks, evidence generation, regulation and market access, workforce readiness, and public trust. Arrows indicate cascade effects where weaknesses in one domain propagate to the next. Overlapping segments represent interdependence between adjacent domains. The central circle conveys the core thesis: when the cycle is intact, AI can reduce health inequalities; when broken, disparities amplify. The directional cycle illustrates cascade effects between domains; in practice, domains also interact directly and non-sequentially. Source: Author’s own creation.

The governance challenges examined here apply to AI technologies broadly, including imaging algorithms, decision-support tools, and predictive analytics. However, we focus specifically on large language models (LLMs) in our discussion of evidence generation, seeing as their stochastic properties expose evaluation gaps that existing frameworks are ill-equipped to address. This perspective argues that solutions lie not only in ‘better’ or ‘smarter’ AI but in health systems whose governance is adapted for the AI era. We organise the perspective around five interconnected domains, drawing selectively on literature identified through the authors’ domain expertise rather than a systematic review: legal frameworks, evidence generation, regulatory and market access mechanisms, workforce readiness, and public trust (see Fig. 1). These domains are interdependent, seeing as gaps in data governance compromise evaluation, inadequate evaluation weakens regulation, and workforce unpreparedness undermines trust. The main contribution of this perspective is found in the integrative framing: while individual challenges have been examined in isolation, they form a governance chain that must be addressed at the health system level, which is explored here. It is worth noting that, while Fig. 1 depicts this chain as a directional cycle to illustrate how weaknesses cascade, the five domains also interact directly with one another in ways that defy a strictly sequential reading; legal frameworks and regulation, for instance, shape each other without requiring mediation by evidence generation, just as public trust bears directly on regulatory legitimacy.

## Finding adaptive governance models for AI equity

A health system refers to the organised arrangements through which healthcare is financed, delivered, and governed, encompassing regulatory bodies, providers, payers, data infrastructures, the workforce, and citizen engagement mechanisms^9^. In this perspective, the argument predominantly centres on macro-level health system governance, where legislative authorities, regulatory decision-makers, and resource allocation converge, while acknowledging that failures at the macro-level cascade across the meso- (e.g., health provider networks, hospital systems) and micro-levels of health systems (e.g., individual clinicians and patient outcomes)^10,11^. It is worth noting that some of the governance challenges discussed in this perspective, particularly those relating to cross-border data-sharing, digital colonialism, and global health data poverty, extend beyond the reach of individual national-level actors and may require transnational coordination mechanisms. There is a growing body of literature that seeks to warn about the potential for AI technologies to worsen inequalities in society. This occurs by distorting citizens’ ability to make informed decisions and by generating inaccurate health information, which are classical failures in principal-agent theory and consequences of imperfect information^6^. Critically, this frames the problem primarily as informational: the ‘informed’ component of informed consent. Yet algorithmic governance may equally compromise the ‘free will’ condition, seeing how AI systems can manipulate choice architectures and pre-empt preferences through behavioural nudging in ways that undermine voluntary agency even when information is complete^12^. Regulators must therefore address not only whether AI improves what patients know, but whether it preserves their capacity for autonomous choice, which is ultimately a normative political decision about the acceptable scope of algorithmic influence in health decisions. Access issues underpinning inequalities may also be worsened if AI technologies are restricted to subsets of a population or through health data poverty (i.e., the diminished ability to benefit from health innovations due to under-representation in health datasets)^6,13^; thereby risking the exacerbation of existing digital and health inequalities^14,15^. Improving representativeness in datasets may not be sufficient either. Even representative data can encode historical and structural biases, thereby enshrining ignorance, if the algorithmic design and outcome definitions lack normative oversight. Ensuring fairness requires attention to the deeper social meanings of categories like race, gender, and disability, and how they are factored into algorithms^16^.

*Within* health systems, AI technologies have the potential to enhance diagnostic accuracy and health service accessibility, which can reduce existing inequalities and improve the personalisation and cost-effectiveness of care (compared to current standards)^7,17^. *Between* health systems, current debates remain largely situated within high-income country contexts, while the implications of AI for health systems in low- and middle-income countries (LMICs), where data infrastructures are often limited and digital health pilots are proliferating, remain critically underexplored. This risks amplifying digital colonialism (i.e., the extraction of data and imposition of digital technologies by high-income countries on low-resource settings, replicating historical colonial patterns), whereby LMICs can become testing grounds for AI tools developed in and for wealthier settings, often without appropriate oversight, accountability, or local benefit^6^; a lesson we can learn from the conduct of randomised controlled trials^18^. Without a governance framework identifying where health systems can intervene, AI will most likely follow the path of least regulatory resistance, concentrating benefits where they are least needed.

## Legal landscape for health data and AI

AI technologies are trained on datasets comprising text, images, or audio. By extension, AI technologies trained on poor-quality datasets are unlikely to meet effectiveness thresholds. More troublingly, modern AI technologies suffer from health data poverty, whereby not all population groups are represented by high-quality data in training datasets, resulting in unwarranted variation in performance^19,20^. Simply increasing representativeness in health datasets does not guarantee that health datasets become more accessible^19^, because of a likely shift towards restrictive licensing and maintaining trade secrets^21,22,78^, which treat datasets as assets from which economic value is derived^22,23^. To address this, laws and regulations must incentivize the findability (the ease with which data can be discovered and located), accessibility, interoperability, and reusability of data following the FAIR guiding principles^24^. Calls for greater data representativeness must, however, be balanced against data sovereignty. LMICs may seek to retain control over health data assets rather than contribute to datasets from which they derive limited benefit, echoing longstanding critiques of extractive research^25,26^. Communities that have experienced structural violence from medical institutions may exercise a right not to be included, and data protection frameworks in some jurisdictions enshrine a right to be forgotten. The challenge is therefore not to maximise inclusion but to ensure governance structures that enable meaningful consent, community control, and equitable benefit-sharing.

One example to improve the findability and accessibility of health datasets is found in the European Union, where the Regulation on the European Health Data Space (EHDS) was ratified in March 2025. This regulation established the requirement for health datasets to be made available for reuse for a number of specific purposes, including the training and development of AI technologies that benefit citizens and patients^27^. The regulation also mandates that some baseline characteristics of each dataset are made openly available in order to increase the findability, accessibility, and interoperability of health data for data pooling and large-scale research^28^. That said, even promising initiatives like the EHDS must complement emerging AI-specific regulatory regimes to address health inequalities. The EU’s AI Act, for instance, introduces a tiered risk-based approach to AI governance, with many health applications expected to fall into the high-risk category by virtue of how the AI Act interacts with the Medical Devices Regulation^28,29^. This leaves equity-related questions within and between these major frameworks unresolved: How will these frameworks interact with existing health data governance to ensure inclusive representation? How will they accommodate real-time model updates and algorithmic drift without exacerbating disparities in safety and effectiveness across different population groups? And how will transparency and accountability be designed to protect those most at risk of harm? Far closer integration between AI regulation and health system governance, grounded in interdisciplinary collaboration among data scientists, ethicists, clinicians, regulators, and citizens, is needed to close these gaps. Without alignment, basic conditions for regulatory innovation may fall short. Moreover, accessible datasets alone are insufficient. How evidence about AI performance is then generated, evaluated, and reported across diverse populations remains a critical determinant of whether these technologies can achieve equitable implementation. Finally, while legal and regulatory developments such as the EHDS may improve the health data ecosystem within the EU, they equally risk widening health data gaps with LMICs if data-sharing provisions are not embedded in a timely manner as part of the EHDS implementation^30^. Legal frameworks for health data are necessary but insufficient by themselves; their value depends on alignment with evaluation standards, regulatory requirements, and international data-sharing arrangements.

## Evidence generation pipeline

The level and quality of evidence supporting medical AI technologies are vital determinants of regulatory approval, market access, and reimbursement decisions. A recent scoping review of reporting gaps in FDA-approved AI medical devices found that reporting of demographic composition in AI medical device studies is inconsistent. Among 692 FDA-approved AI medical devices reviewed, less than 20% reported the age distribution of the study population, 3.6% reported on race/ethnicity distribution, and 1% provided socioeconomic details^31^. Complementing these reporting deficits, a scoping review of 467 clinical AI fairness studies found that fairness research remains concentrated in a small number of bias-relevant attributes, with over half of the included studies examining only race/ethnicity or gender/sex as bias-relevant attributes and few considering socioeconomic status, language, or intersectional effects^32^.

**Fig. 2:**
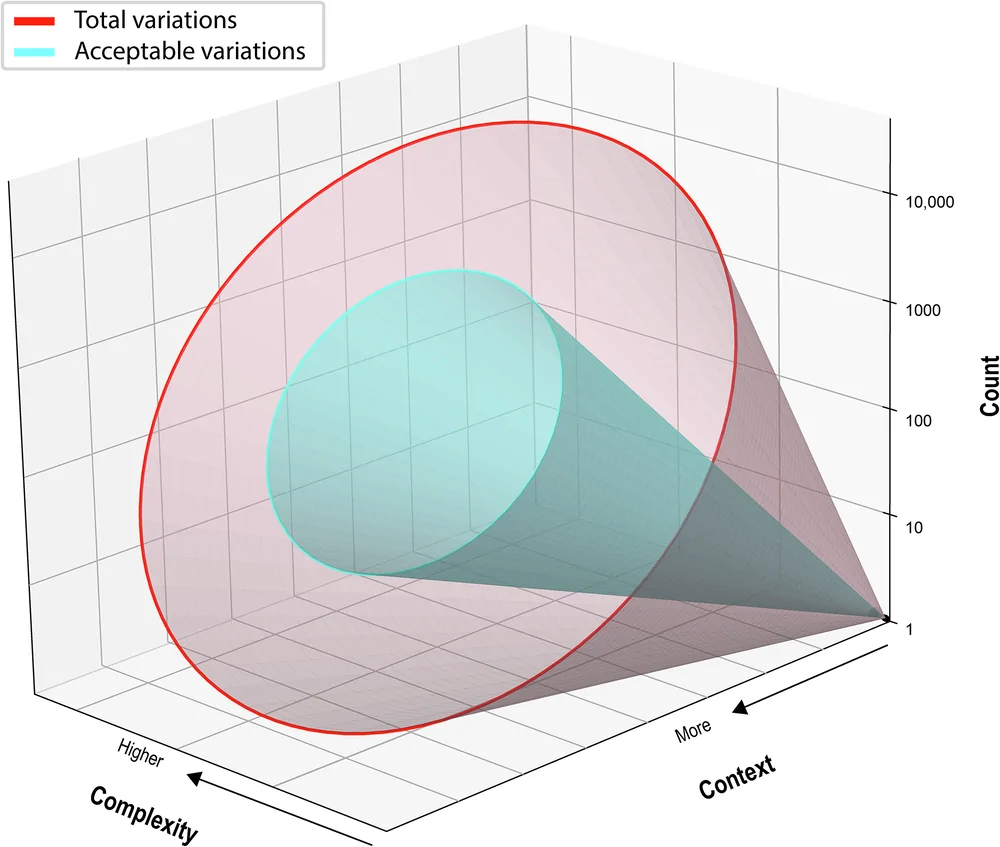
An illustrative 3D visualisation of acceptable versus total variations in LLM outputs. This illustrative example shows how the acceptable variation (i.e., the normative assessment of what are contextually appropriate outputs) and total variation (i.e., the full distribution of outputs generated across repeated sampling) of LLM outputs increase depending on the contexts of deployment (x-axis), complexity of the situation (y-axis), and count of outputs generated (z-axis). The origin point of both cones is positioned at the corner of axes representing the health system context and the number of LLM outputs, representing the generation of a single output in a single health system context of low complexity. Variation expands outward as the number of contexts, case complexity, output count increase. Note: the log-linear increase of the total and acceptable variation is illustrative, and real-world assessments of LLMs may yield other non-linear relationships between total and acceptable variation. Source: Author’s own creation.

In the context of LLMs, evaluations tend to take an N-of-1 approach, ignoring the stochastic nature of these technologies. These evaluations frequently use one or more scenarios, where each scenario contains the same list of questions. However, for each scenario-question combination, only a single LLM output tends to be generated^33–39^, which does not capture acceptable and unwanted variations in LLM outputs within and between population groups (see the illustrative example in Fig. 2). Especially when LLMs are trained to be deployable in multiple health system contexts and for situations of varying medical complexities, understanding what variations of LLM outputs are acceptable is a critical step in developing the safety profile of the technology while retaining the ability to leverage the generative capabilities of LLMs^40^. The current N-of-1 evaluations yield important yet preliminary insights into the performance and risk profiles of LLMs. These evaluations should instead be treated as gateway evidence to secure funding for larger-scale research that is able to capture divergent LLM performance across population groups. When evaluations do not characterise performance across demographic subgroups, disparities in accuracy and safety pass undetected through the evidence-to-deployment pipeline, meaning regulatory and reimbursement decisions may rest on evidence that is structurally blind to the populations most at risk.

Moreover, the limitations of current evaluation paradigms are compounded by well-documented risks inherent to LLMs. These include hallucinations, in which models generate confident but factually incorrect medical statements^33,41^; misalignment between user intent and model output that can yield unsafe, or context-inappropriate outputs^42,43^; and limited explainability, which obscures the reasoning pathways driving LLM-generated recommendations^44^. Each of these risks poses distinct clinical threats and challenges the foundations of the existing medical evidence hierarchy, which assumes consistent, transparent, and mechanistically interpretable interventions. Multi-output, population-sensitive evaluations are essential not only to characterize model performance but also to manage wider risks.

Health systems host sophisticated regulatory and technology assessment mechanisms for high-risk health technologies, which have started to evolve in order to capture AI technologies as well^28^^,56^^,78^. As the evolution of these mechanisms for AI technologies is ongoing^17^, researchers can start to improve the evidence pipeline of medical AI technologies. Different clinical trial designs can help capture real-world costs and performance of medical AI technologies (see Table 1)^45–49^, so long as appropriate and meaningful end-points are well-defined in advance^50^. Health economic evaluation methods can subsequently use this data to inform whether the costs are proportional to the benefits derived, and how those benefits are distributed across population groups^51^. Suitable end-points for AI-focused health research are yet to be defined and formalized, which is a weakness in the regulatory approval and health technology assessment (HTA) process^50,52^.

**Table 1:** Overview of the alternative study designs and their application to AI technologies

Study Design	Deviation from traditional RCT design	Benefits for AI technologies
Cluster randomized study	Cluster RCTs randomize groups (e.g. clinics, hospitals, communities) instead of individual participants to different intervention arms.	• Suited for AI technologies supporting group efforts. • Useful when the intervention consists of multiple individual elements that interact with each other • Direct and indirect effects of an intervention can be evaluated.
Micro-randomized study	Micro-randomized studies randomize interventions multiple times per participant (e.g., daily or hourly) to evaluate the short-term effects of just-in-time adaptive interventions.	• Suited to determine the efficacy of a specific component of AI technologies. • Usable especially in the early stages of a product development.
Pragmatic trial	A pragmatic trial is an RCT conducted under typical real-world conditions rather than tightly controlled settings. Pragmatic trials use broad inclusion criteria and embed the intervention into routine care with minimal extra monitoring or protocol-mandated restrictions. Blinding may be relaxed, and multiple real-world sites may be involved.	•Yields high external validity and real-world clinical effectiveness data. •Demonstrates how AI technologies perform in diverse patient populations and workflows. •Supports cost-effectiveness through the use of existing infrastructure and representative outcomes.
Observational trial	No randomization; researchers observe outcomes as they occur in practice. This includes prospective cohorts, registries, or retrospective EHR analyses. Higher risk of confounding compared to RCTs.	• Provides real-world evidence of effectiveness and cost-effectiveness at scale. • Useful for assessing AI technologies using EHR or claims data. • Reflects actual use and outcomes in broad populations.
Target trial emulation	Emulates a hypothetical RCT using observational data. Defines eligibility, interventions, outcomes, and follow-up as if conducting an RCT, but without randomization. Reduces bias through protocol-like structure and confounding adjustment.	• Enhances credibility of real-world evidence. • Allows assessment of AI technologies post-deployment using large-scale datasets. • Faster and cheaper than prospective trials while approximating causal inference.
Zelen’s design	Randomizes patients before consent. In the single-consent version, only the intervention group is informed and consents. The control group gets standard care, often without knowing they were in a study. Intention-to-treat analysis applied.	• Simplifies recruitment and reflects the routine care offer of digital tools. • Minimizes disappointment and contamination bias. • Suitable for low-risk interventions with variable adherence. May support generalizability and policy relevance.
Silent trial	Silent trials refer to prospective, noninterventional assessments of AI models in the intended setting without affecting patient care or institutional workflow.	• Suitable for assessing whether the model will maintain its performance in a live context using local evidence. • Allows testing for potential utility (e.g., pipeline stability, model drifts), as well as financial sustainability of models in real world evaluations without affecting the clinical workflow.

For LLMs specifically, moving beyond the N-of-1 paradigm requires repeated-sampling designs to characterise output variability, pre-specified demographic subgroup analyses with minimum reporting standards, acceptability thresholds for performance variation defined a priori, and post-deployment monitoring with equity-specific endpoints. These should be structural requirements of regulatory and HTA processes, not optional additions. Furthermore, it is worth noting that some of these requirements, especially around sampling and subgroup analysis, are already known practices within health systems^53,54^. These practices should be leveraged as a starting point to create LLM-specific guidance for high-quality evidence generation.

Post-market surveillance and the ability to monitor AI model drift, performance degradation, or updates over time remain critical gaps in current regulatory and clinical practice^55^. Unlike traditional medical technologies, many AI systems, particularly those employing dynamic or continuously learning algorithms, can evolve after deployment, introducing new risks to safety, efficacy, and equity. Yet most existing regulatory frameworks are designed for static technologies and do not account for the need to revalidate or reassess AI tools as they change^56^. Compounding this challenge is the so-called “black box” problem: the opacity of deep learning systems can make it difficult to understand how outputs are generated and what alternative outputs were possible^57^. This lack of transparency poses serious regulatory and ethical risks, particularly when AI-generated recommendations are made in the absence of a clear pathway for clinicians to retrace or challenge the reasoning behind them. These issues underscore the urgent need for adaptive regulatory mechanisms, robust auditability protocols, and real-world performance monitoring with agreed endpoints.

The environmental costs of AI compound these equity concerns^58^. The energy-intensive nature of training and deploying large-scale AI models, especially foundation models, poses ethical, resource allocation, and population health challenges, particularly for health systems operating under climate constraints or energy scarcity^59,60^. Integrating climate impact into evaluation frameworks is therefore integral to, not separate from, the equity agenda. In sum, the evidence pipeline is where governance determines whether AI is held to equity-sensitive standards or advances on evidence blind to at-risk populations.

## Regulatory mechanisms and market access incentives

Existing medical device and clinical trial regulations include provisions for demographic representation in clinical trials (e.g., FDA Safety and Innovation Act Section 907 in the USA, and the EU Clinical Trials Regulation)^61,62^, which apply to AI when classified as a medical device. However, no AI-specific provisions address the distinct challenges of representativeness in training datasets and algorithmic evaluation, where biased data, opaque architectures, and context-dependent performance variation compound in ways traditional requirements were not designed to capture. Lessons from other domains suggest that combining regulatory obligations with targeted incentives may be particularly effective. For example, several recommendations have been made to increase the representation of women in health research, including granting regulatory bodies permission to mandate the inclusion of women in clinical trials and obligating the disaggregation of clinical data between men and women^63^. Such approaches recognize that adequate representation is not a discretionary feature but a fundamental prerequisite for safe and effective medical technologies. Beyond these baseline obligations, additional incentive mechanisms could reward exemplary efforts that go substantially beyond minimum requirements^63,64^. A similar tiered approach could be adapted for AI technologies: minimum representativeness standards would be required for market access and reimbursement, while enhanced incentives could be reserved for developers who demonstrate exceptional commitment to equity, such as addressing historically underserved populations, conducting prospective evaluations of performance across intersectional subgroups, or developing AI systems specifically designed to reduce rather than replicate existing disparities. This combined approach would ensure that inclusive development is not optional while still encouraging innovation that actively advances health equity.

The success of regulation and incentives rests on understanding markets. The substantial contribution of the venture-capital-driven model for AI development is informative, suggesting a market driven by rapid monetization and commercial scalability. This may be at odds with health system objectives, including equitable scalability. In addition to creating R&D incentives to steer development, payers and governments can address concerns by reforming payment models away from itemised payments (such as fee-for-service) towards bundled payments that are developed to incentivise high-quality, equitable care that improves population health, thereby rewarding providers for value-based AI use (not simply AI use). To achieve this, clear standards about what constitutes ‘value’ need to be developed and embedded in payment mechanisms. These challenges are amplified in LMICs, where regulatory capacity for AI is limited, and market incentives are less likely to drive development tailored to local disease burdens. Without international coordination on minimum standards and LMIC participation in governance design, market access pathways risk reinforcing global health inequalities. Equally, without equity-sensitive evaluation criteria embedded in regulatory processes, even well-evaluated technologies may be deployed in ways that entrench disparities in access and outcomes.

## Inequalities of human capital and AI training opportunities

While regulatory and market access mechanisms determine which AI technologies reach clinical settings, their equitable implementation depends on whether the workforce is prepared to use them appropriately. Training of health professionals to use AI within their medical practices and workflows has become a known necessity^65,66^. The development of what AI-related skills and competencies are required for health professionals is equally a rapidly advancing field, with consensus on core competencies starting to crystallise^65^. This is especially important given evidence that, in specific task classes such as medical imaging, AI technologies have demonstrated diagnostic accuracy comparable to or exceeding that of clinicians under controlled conditions^1,67^, with potential workflow benefits^68^, though real-world evidence remains limited. However, structural integration of AI training in medical curricula remains inconsistent^65,69^, while the resources required to upskill the current healthcare workforce may not be available at every medical centre or for every healthcare professional (e.g., nurses, general practitioners, specialists may have unequal access to training). Combined, this could have downstream effects of the quality of patient care when AI technologies are either inappropriately used or inappropriately avoided. These risks are magnified in LMICs, where baseline medical education infrastructure is under strain and resources for AI-specific training are most scarce^6,70^.

While the potential for AI to improve disease detection and diagnostic accuracy remains an active area of investigation with context-dependent results, reports are emerging of how overreliance on AI for diagnostics and decision-making can lead to deterioration of clinicians’ core skills, such as critical thinking, diagnostic reasoning, and hands-on expertise, especially if routine or foundational tasks are delegated to AI^71,72^. This deskilling may subsequently result in clinicians being less prepared to act independently when AI is unavailable, malfunctions, or provides erroneous outputs; or when complex or unexpected situations arise, potentially compromising patient safety^71,73,74^. Altogether, disparities in AI literacy risk a two-tier system in which the quality of AI-augmented care depends on where and by whom it is delivered.

## Public trust and legitimacy underpin value

The uptake and impact of AI tools in health systems will depend not only on technical performance but also on whether patients, clinicians, and the public perceive them as useful, trustworthy, fair, and transparent^28,75,76^. This trust is not formed in isolation, but rather depends in part on whether the evaluation, regulatory, and implementation processes discussed in previous sections are perceived as credible and inclusive. Governance models must include mechanisms for democratic oversight and community engagement, ensuring that those most affected by AI interventions have a voice in their design and implementation. In practice, this requires institutional channels, including citizen assemblies, standing patient and public involvement panels, and statutory consultation obligations on regulators, that give affected communities a formal role in shaping which AI tools are approved, how they are deployed, and under what conditions they are withdrawn. Without such inclusive processes, even well-performing systems may lack the social licence for implementation and use^77^. This means that discourse shaping the legitimacy of all AI technologies needs to be addressed as part of AI governance approaches. For example, addressing environmental concerns can augment public trust in, and the value of, AI technologies, particularly at the global level, given that climate change will disproportionately affect those who likely benefit the least from AI technologies^59^. This exemplifies the need for sophisticated governance models that account for global geographic equity concerns between health systems and regional or national concerns relating to equitable access within health systems in the pursuit of building the public trust and legitimacy necessary for AI technologies to bring value to health systems. Public trust is ultimately positioned here as a precondition for governance, not its outcome; without the necessary social licence, the reforms proposed here will lack the legitimacy needed for implementation.

## Conclusion

AI technologies are a 70-year-old overnight phenomenon that is currently finding its place in society. Lofty expectations and grave warnings coexist in the health literature^2^, though conclusive evidence for either side has yet to emerge. This perspective highlights how the evidence-demanding nature of health systems can become an instrumental force in fostering a sustainable and beneficial place for AI technologies that reduces inequalities, while safeguarding and minimising the probability of AI-related harms. Simultaneously, it showcases in each section how, when one part of the governance chain is deprecated or circumvented, AI-related inequalities may worsen (summed up in Table 2).

**Table 2:** Governance domains, gaps, and proposed actions for addressing AI-related inequalities in health systems

Domain	Current gaps	Proposed action	Cost of inaction
Legal frameworks	Fragmented governance; restrictive licensing; limited LMIC data-sharing	Align with FAIR principles; embed equity in EHDS-type frameworks; benefit-sharing agreements	Data poverty persists; AI underperforms for underserved populations
Evidence generation	Poor demographic reporting; N-of-1 LLM evaluations; no equity endpoints	Mandate subgroup reporting; repeated-sampling designs; a priori acceptability thresholds	Technologies approved on evidence blind to at-risk populations
Regulation & market access	No AI-specific representativeness standards; volume-based payment	Minimum representativeness for market access; equity incentives; value-based payment	Benefits concentrated in commercially attractive populations
Workforce	Inconsistent AI curricula; unequal training access; deskilling risk	Systematic competency integration; equitable resourcing; atrophy safeguards	Two-tier care quality by provider training access
Public trust	Limited citizen voice; transparency deficits; environmental concerns	Democratic oversight; environmental impact in evaluation; community engagement	AI lacks social licence; reforms lack legitimacy

To seize this leadership role, health systems must recognise that the governance domains examined in this perspective, from data access through evaluation, regulation, workforce preparation, and public legitimacy, are interdependent and require integrated, adaptive governance. Public trust, transparency, and accountability must be core to this because these are building blocks of value creation. Citizens must have a role in shaping how AI is used in healthcare, not only as data subjects, but as democratic stakeholders. Without inclusive governance, even technically sound systems may lack legitimacy, restricting their innovative potential.

## Data Availability

No datasets were generated or analysed during the current study.
